# Supplementary material for: Chromothripsis during telomere crisis is independent of NHEJ, and consistent with a replicative origin
Source: Genome Res. 2019 May;29(5):737–49. doi: 10.1101/gr.240705.118 (PMC6499312; doi:10.1101/gr.240705.118)
Supplement: Supplemental Material [file supp_gr.240705.118_Supplemental_file_1.zip › contigs/annotated_contigs/DB45/contig.2.DB45_length_694_mean_cov_6.08645533141.docx]

**DB45_length_694_mean_cov_6.08645533141**

```
TGTGCCACGTTGGTGTGCTGCACCCAGTAACTCGTCATTTAACATTAGGTATATCTCCTAATGCTATCCCTCCCCACTCCTCCCACCCC
 >chr3:105344338-105344710 - E=5e-138
ACAGCAGGCCCTGGTGTGTGATGTTCCCCTTCCTGTGTCCATGTGTTCTCATTGTTCAGCTCCCACTTATGAGTGAGAATATGCGGTGT

GTGGTTTTCGGTTCCTGTGTTAGTTTGCTGAGAATGACGGTTTCCACCTTCATCCATGTCCCTGCAAAGGACATGAACTCATCCCTTTT

ATGGCTGCATAGTATTCCATGGTGTATTTGTGCCACATTTTCTTAATCCAGTCTATCATTGTTGGACATTTGGGTTGGTTCCAAGTCTT

TGCTATTGTG|AATAG|AATGGATGTTCAAAGCTTGCAAAATTTTCCAAAAATTTTATACCAAATAAATAGTTGCATAATACCCAAACT
           >chr2:193855880-193856208 + E=1e-179
AATTTCCCTAGATTACCAATAGCTTTTCTACCCCTTCATTCAGACATGAAATGAAAGAAAAGAAAGGTAAAATTATTAAACAGTTTCAA

ACACAAAATACCTGGTTTACTTTCAGGTAGAATTATAAACATAAATATTTTTATGACTATAATGTGACATTTCCTCTTACTTTAGAGTA

GAATAAAAAATACAACCTTCCCCAGCTGAACTAGTGAAGTTAAATGCATGTCAATACTGTCCTCCTAGCTGAA
```
